# Ignored and distressed: a cross-sectional study of the impact of COVID-19 on last responders

**DOI:** 10.1186/s12889-023-16565-z

**Published:** 2023-08-26

**Authors:** Rima A. Afifi, Jorge Luis Calderon, Hanh Pham, Peter Teahen, Sydney Zarate-Sada, Daniel K. Sewell, Mark W. Vander Weg

**Affiliations:** 1 Department of Community and Behavioral Health, College of Public Health, University of Iowa, 145 N Riverside Drive, Iowa City, Iowa, N416 USA; 2 Department of Biostatistics, College of Public Health, University of Iowa, Iowa City, Iowa, USA; 3 Teahen Funeral Home, Cedar Rapids, Iowa, USA; 4 Clinical Mental Health Counseling, College of Education, University of Iowa, Iowa City, Iowa, USA

**Keywords:** COVID-19, Mental health, Last responders, Stigma

## Abstract

**Background:**

Last responders constitute an occupational category that includes all those that are involved in the postmortem care of deceased persons and their families. Last responders are exposed to several categories of work-related stressors that affect their health and well-being. COVID-19 exacerbated these stressors. Research to understand the consequences of COVID-19 on the health and wellbeing of last responders is nascent. This study aimed to assess COVID-19 related stress, coping and wellbeing among last responders in the United States.

**Methods:**

We conducted a national cross-sectional survey of last responders in July through September of 2020. The survey measured wellbeing, stress, coping, and stigma; COVID-19 experiences, and socio-demographics. A ridge regression model was fit for the outcome variables.

**Results:**

Analysis was conducted on 366 respondents from 43 states. Respondents were male (55.4%), age 50 + (57.4%), and White non-Hispanic (90.3%); 54% reported moderate-high stress and 41% endorsed mild-severe anxiety. Seventy-seven percent had experienced at least one form of stigma related to their occupation. Variables associated with higher perceived stress and anxiety included gender (female), shorter length of employment, perceiving a higher impact from COVID-19 on everyday life, and increased perceived stigma.

**Conclusions:**

Last responders are a critical part of the health care system. Throughout this pandemic, last responders have been frequently ignored and not prioritized for protection and support. Interventions to support last responders cope with stress, and to decrease anxiety are urgently needed. There is also a critical need to challenge community stigma towards last responders.

**Supplementary Information:**

The online version contains supplementary material available at 10.1186/s12889-023-16565-z.

## Background

Last responders constitute an occupational category that includes all those that are involved in the postmortem care of deceased persons and their families. Although managing the complex reality of preparing the body and coordinating the burial event causes strain, [1] there has been little research into work-related stress and mental health of last responders [2].

Last responders are exposed to several categories of work-related stressors. These stressors include factors that also affect others in health-related professions such as managing shift work and work-life balance [3]. Stressors also include those specific to the job of a last responder, such as working in a non-traditional job, constant exposure to deaths, and dealing with bereavement [3]. Previous report indicate that these stressors affected mental health of last responders directly, and indirectly through perceived stress [3]. Forty-three percent of funeral directors in the United States (US) have considered leaving their profession because of continued exposure to death [4].

The wellbeing of last responders is also related to the manner of death of persons for whom they are caring. Traumatic death circumstances cause a significant amount of stress, can overwhelm the coping responses of last responders, and cause mental health distress and emotional exhaustion [5, 6]. Work-related stress can produce frustration, feelings of isolation, concern over physical health, apathy, irritability, and fear of abandonment – all affecting wellbeing [5].

Some of the research around last responder stress and mental health is reported from military experience with mass casualties. Dealing with the dead in war situations is difficult as a result of the large number of casualties; sensory stimuli, particularly smell; disenfranchised grief; and stigma [6]. In mass casualties’ situations, coping strategies included reaching out to coworkers for support [6]. Social support from their families is rare as last responders choose not to share what they experienced with their families, so as to ‘protect’ them from the trauma and stress [6].

The COVID-19 pandemic began in the US in January 2020. The scope and scale of resulting deaths can be considered akin to a mass casualties situation. Last responders have experienced significant increases in exposure to death. As of March 24, 2023, there have been over 1,123,610 deaths that had been attributed to COVID-19 in the US [7]. Last responders are also at the front lines of the COVID-19 pandemic. This adds to the potential stress of the job as they may be exposed to the virus, possibly putting themselves and their families at risk [2]. Depression and anxiety symptom levels were found to be elevated among Canadian funeral service workers during the early stages of the pandemic [8]. Anecdotal evidence suggests that COVID-19 may mimic other aspects of mass casualties situations, including disenfranchised grief, and stigma. In the US, the topic of death remains taboo and stigmatized [6, 9]. Traumatic stress events, number of funerals, high demands of the job, and overexposure to death during their work are purported to lead to negative mental health impacts [2].

Last responders have generally been ignored in the COVID-19 pandemic, despite significant changes (increasing deaths, concerns about COVID-19 deaths, inability to engage families, disruptions in the traditional grieving process) in the job for many of them [10]. Research to understand the consequences of COVID-19 on the health and wellbeing of last responders is nascent, but critically important to development of effective responses [2]. This study aimed to assess COVID-19 related stress, coping and wellbeing among last responders in the US, and is the first national survey to assess stress and mental health of last responders as an occupational category.

## Methods

### Participants

The data for this study were obtained from a national cross-sectional survey of last responders. The survey was distributed in July–September 2020 through 9 last responders associations (e.g., the National Funeral Directors Association, the National Association of Medical Examiners—the full list of associations is included in the acknowledgements section). We asked executive directors or presidents of these associations to send an invitation to the survey to their membership. This invitation letter included general information about the purpose of the survey. Two reminder invitations were sent, each one-week part from the original invitation to encourage participation. A link to the online survey was imbedded in the invite for those interested in participating. Eligibility included being a last responder in the US, being over 18 years of age, and not having completed the survey previously. The survey closed on September 30 2020.

### Instrument

The survey instrument consisted of validated measures of wellbeing, stress, coping, and stigma; and items measuring COVID-19 experiences and perceptions, as well as socio-demographics. The survey and study procedures were approved by the Institutional Review Board (IRB) at the University of Iowa. The first page of the survey was an informed consent statement; continuing on to the survey indicated consent to participate. Respondents could complete the survey from receipt of the invitation to participate until the survey was closed at the end of September 2020; 535 respondents provided sufficiently complete survey responses. Last responders could be members of more than one of the associations that distributed the invitation letter. Also, last responders who received the invitation were invited to share it with others who fit the eligibility criteria and were interested, even if outside of these associations. For these two reasons, we are unable to calculate a response rate because we do not know the denominator.

### Survey variables

We only describe here the survey variables used in the current analysis.

#### Socio-demographics, and Work Experience

Socio-demographic variables included sex, age, race/ethnicity, and educational level. We also asked respondents if they had any pre-existing health conditions. Our survey included the following variables related to their work experience: number of years employed as a last responder, and indicators corresponding to having had previous experience with infectious diseases, having had previous experience with mass fatalities, and having handled a suspected COVID-19 related death. We asked about the State in which they worked.

#### Well-being (WHO-5, GAD-7)

Overall well-being was measured using the World Health Organization’s Well-Being Index (WHO-5), responses range from 0 and 25 with higher values indicating better well-being [11]. Anxiety was measured using the Generalized Anxiety Disorder Scale (GAD-7), responses range between 0 and 21 with higher values indicating more anxiety. Cut off points for minimal (0–4), mild (5–9), moderate (10–14), and severe (15–21) anxiety have been identified [12].

#### Stress (Perceived Stress)

We used the 10-item Perceived Stress Scale (PSS) [13]. PSS responses range between 0 and 40 with higher values indicating more stress. Cut off points have been identified for low (0–13), moderate (14–26), and high (27–40) stress.

#### Coping (brief COPE)

We used the 28-item Brief-COPE questionnaire [14], which includes 14 subscales which can be collapsed into two broader subscales: adaptive and maladaptive. The adaptive coping subscale is the sum of subscales 1–8, and the maladaptive coping subscale is the sum of subscales 9–14 [14]. This grouping was verified through visual inspection of clear clustering patterns in a principal components analysis bi-plot.

#### Social support (FSSQ)

Social support was measured using the Functional Social Support Questionnaire (FSSQ) [15]. FSSQ values range from 1 to 5, with higher values indicating greater amount of perceived social support.

#### Stigma

We used the Everyday Discrimination Scale as a measure of stigma [16]. This scale includes 9 items related to experiences of discrimination as a result of some characteristic, we inquired about their experience with these items in relation to their occupation as a last responder. Responses options range from never (1) to often (4). We created a stigma score which was the average of the responses to the 9 items.

#### COVID-19 related survey items

We included several COVID-19 related questions. These variables measured whether the respondent had COVID-19, whether a member of the respondent's’ family had COVID-19, whether COVID-19 affected the respondent’s life or job, whether the respondent had sufficient personal protective equipment (PPE), whether the respondent did not use PPE due to social pressure, and whether the respondent felt anxiety over PPE shortages.

The full survey is included as an Additional file.

### Data analysis

A ridge regression model was fit for the outcome variables well-being, anxiety, stress, and coping. Social support, stigma, and COVID-19 related survey questions were included as covariates in each model as well as socio-demographic variables. Because a preliminary analysis revealed a highly positive correlation between worker's confidence in protecting themselves against COVID-19 and their confidence in protecting their family against COVID-19 (*r* = 0.75, *p* < 0.01), we only included the latter covariate.

In addition to the survey questions, in each outcome’s regression model we included excess mortality during the pandemic in the State in which the respondent worked, as a covariate. This variable accounts for the fact that some last responders reside in the states that experienced a disproportionately higher death toll throughout the pandemic, while adjusting for between-state variation in reporting COVID-19 as cause of death. The estimated observed and expected total counts of death for each state between the beginning of the pandemic, January 2020, and the date each person took the survey was obtained from the National Center for Health Statistics website. The proportion increase in death was calculated as the difference between the observed and expected number of deaths divided by the expected.

The shrinkage parameter in the ridge regression was selected via grid-search over a range of values to minimize mean cross-validation error. Inferential results were based on 95% bias-corrected and accelerated bootstrap confidence intervals. Due to the large number of covariates, only those that have significant association with an outcome in each model are reported in the Results section.

We retained for our analyses all subjects who were missing 25% or less of the variables. To handle the remaining missingness in the data, we used multiple imputation with 10 imputed datasets, and combined this with our bootstrap-based inference using the MI BOOT algorithm in Schomaker and Heumann (2018) [17].

## Results

There were 366 respondents with 25% or fewer missing values on anxiety, wellbeing, perceived stress, and all covariates (Table 1). Forty-three states were represented. The majority of respondents were male (55.4%), age 50 + (57.4%), and White non-Hispanic (90.3%). The average (sd) reported length of work was 22.40 (13.98) years. Most last responders had some level of experience with handling infectious deaths (89.1%) but only 34% previously worked with mass fatalities. Fifty-four percent of last responders reported moderate to high stress and 41% endorsed mild, moderate, or severe anxiety. The average (sd) score perceived stress was 14.81 (7.35), for anxiety was 5.18 (5.63), for wellbeing index was 13.11 (6.08). Generally, last responders utilized more adaptive coping strategies (e.g., active coping, acceptance, positive reframing) than maladaptive ones (e.g., self-distraction, denial, venting). The adaptive and maladaptive coping subscales were only weakly correlated (*r* = 0.38, *p* < 0.01). Seventy-seven percent of last responders had experienced at least one form of stigma related to their occupation. The average (sd) stigma score was 1.56 (0.56).

**Table 1:** Demographics characteristics of last responders

	N^b^	Range	n (%) / mean ± sd
**Sex**	363		
Female			162 (44.6)
Male			201 (55.4)
**Age group**	364		
20–29			19 (5.2)
30–39			61 (16.8)
40–49			75 (20.6)
50–59			103 (28.3)
60 +			106 (29.1)
**Race ethnicity**	359		
White non-Hispanic			324 (90.3)
Other			35 (9.7)
**Education level**	363		
HS/GED/Technical degree			98 (27.0)
Undergraduate			144 (39.7)
Graduate			121 (33.3)
**Work hour**	364		
Part-time/Unemployed			15 (4.1)
Full time			349 (95.9)
**Work length in years**	365		22.40 ± 13.98
**Previous experience w. infectious diseases**	359		
A lot			128 (35.7)
Some			192 (53.5)
Not at all			39 (10.9)
**Previous experience w. mass fatalities**	366		124 (33.9)
**Handled COVID-19-suspected death**	366		302 (82.5)
**Has any pre-existing health condition**	366		133 (36.3)
**Family member had COVID-19**	360		
Yes			36 (10.0)
No			299 (83.1)
Unsure			25 (6.9)
**Self had COVID-19**	366		
Yes/Unsure			76 (20.7)
No			290 (79.2)
**I had enough PPE to protect me**^**a**^	343	1–5	3.90 ± 1.42
**I didn't use PPE because of PPE shortage**^**a**^	341	1–5	1.59 ± 1.12
**I didn't use PPE because of social pressure**^**a**^	334	1–5	1.31 ± 0.81
**Shortage of PPE makes me anxious**^**a**^	339	1–5	3.18 ± 1.54
**Able to protect family from COVID-19**^**a**^	365	1–5	3.42 ± 1.12
**Impact of COVID-19 on life**	365		
Not at all / Somewhat			197 (54.0)
Very much			168 (46.0)
**Impact of COVID-19 on job**	362		
The same/Easier			74 (20.4)
Harder			288 (79.6)
**Stigma score**	364	1–4	1.56 ± 0.56
**Stigma Categories**	364		
At least one form of stigma			281 (77.2)
Never experienced any stigma			83 (22.8)
**Functional Social Support Questionnaire**	366	1–5	4.34 ± 0.76
**State-level excess mortality (proportion)**	366		0.14 ± 0.09
**General Anxiety Disorder-7**	363	0–21	5.18 ± 5.63
**General Anxiety Disorder Categories**	363		
Minimal		0–4	213 (58.7)
Mild		5–9	80 (22.0)
Moderate		10–14	32 (8.8)
Severe		15–21	38 (10.5)
**WHO-5 Well-being Index**	363	0–25	13.11 ± 6.08
**Perceived Stress**	366	0–40	14.81 ± 7.35
**Perceived Stress Categories**	366		
Low		0–13	169 (46.2)
Moderate		14–26	166 (45.4)
High		27–40	31 (8.5)
**Adaptive Cope**	272	16–64	38.31 ± 8.83
**Maladaptive Cope**	297	12–48	20.12 ± 6.08

^a^Variable measured on a Likert scale with 1 = Strongly Disagree and 5 = Strongly Agree

^b^Number of respondents with complete data for the corresponding variable

### Well-being

Table 2 summarizes the results of the two ridge regression models predicting anxiety and wellbeing. Variables that have a significant association with both decreasing anxiety and increasing well-being include gender (male), more years in the profession, higher confidence in the ability to protect family members from COVID-19, lower stigma scores, and stronger perceived social support. Other factors associated with decreasing anxiety include having enough PPE, less concern about PPE shortage and perceiving a lower impact from COVID-19 on everyday life. Additional factors having a significant association with increasing well-being include being unsure if family members had COVID-19 infections, perceiving a lower impact of COVID-19 on the job, and lower state-level excess mortality rate.

**Table 2:** Results of significant variables in either of the regression models predicting anxiety or well-being

	**General Anxiety Disorder-7**		**Wellbeing Index**	
	Coefficient	95% CI	Coefficient	95% CI
**Sex**				
Female (reference)	-	-	-	-
Male	-1.06	(-1.83, -0.28)	0.99	(0.07, 1.91)
**Work length in years**	-0.06	(-0.09, -0.03)	0.08	(0.04, 0.11)
**Family member had COVID-19 **				
Yes (reference)	-	-	-	-
No	-0.46	(-1.69, 0.76)	0.64	(-0.68, 1.96)
Unsure	-0.84	(-2.52, 0.83)	1.93	(0.11, 3.75)
**I had enough PPE to protect me**^**a**^	-0.29	(-0.57, -0.01)	0.17	(-0.14, 0.48)
**Shortage of PPE makes me anxious**^**a**^	0.34	(0.08, 0.59)	-0.29	(-0.60, 0.01)
**Able to protect family from COVID-19**^**a**^	-0.79	(-1.18, -0.41)	0.62	(0.24, 1.01)
**Impact of COVID-19 on life**				
Not at all / Somewhat (reference)	-	-	-	-
Very much	1.74	(0.94, 2.54)	-0.69	(-1.62, 0.24)
**Impact of COVID-19 on job**				
The same/Easier (reference)	-	-	-	-
Harder	0.31	(-0.54, 1.16)	-1.28	(-2.43, -0.13)
**Stigma score**	1.91	(1.08, 2.74)	-1.84	(-2.60, -1.08)
**Functional Social Support **	-0.75	(-1.35, -0.16)	1.30	(0.67, 1.93)
**State-level excess mortality (proportion) **	2.37	(-1.82, 6.56)	-5.22	(-10.29, -0.14)

^a^Variable measured on a Likert scale with 1= Strongly Disagree and 5=Strongly Agree

### Perceived stress

Table 3 shows the results of significant covariates in the model predicting stress level. All the significant variables in both the anxiety and wellbeing models are also significant in the stress model, as well as age, anxiety over PPE shortage, and the perceived impact of COVID-19 on one’s everyday life and job. Variables associated with higher perceived stress are gender (female), being in the 20–29 age group vs the 50–59, shorter length of employment, anxiety over PPE shortage, lower confidence in the ability to protect family members from COVID-19, perceiving a higher impact from COVID-19 on one’s everyday life and job, increased perceived stigma, and decreased social support.

**Table 3:** Results of significant variables in the regression model predicting stress levels

	**Perceived Stress**	
	Coefficient	95% CI
**Sex**		
Female (reference)	-	-
Male	-1.07	(-2.12, -0.02)
**Age group**		
20–29 (reference)	-	-
30–39	0.38	(-1.06, 1.82)
40–49	-0.05	(-1.41, 1.32)
50–59	-1.38	(-2.63, -0.13)
60 +	-0.64	(-1.73, 0.46)
**Work length in years**	-0.07	(-0.11, -0.03)
**Shortage of PPE makes me anxious**^**a**^	0.49	(0.13, 0.85)
**Able to protect family from COVID-19**^**a**^	-0.69	(-1.14, -0.23)
**Impact of COVID-19 on life**		
Not at all/Somewhat (reference)	-	-
Very much	1.59	(0.56, 2.61)
**Impact of COVID-19 on job**		
The same/Easier (reference)	-	-
Harder	1.15	(0.01, 2.28)
**Stigma score**	2.25	(1.24, 3.25)
**Functional Social Support**	-1.98	(-2.73, -1.24)

^a^Variable measured on a Likert scale with 1 = Strongly Disagree and 5 = Strongly Agree

### Coping

Results of the two models predicting the adaptive or maladaptive coping subscales can be found in Table 4. The use of more adaptive coping mechanisms was associated with more anxiety over PPE shortages, perceiving a higher impact from COVID-19 on everyday life, and higher social support. The use of more maladaptive coping mechanisms was associated with shorter length of employment, lower confidence in the ability to protect family members from COVID-19, perceiving a higher impact from COVID-19 on everyday life, and higher perceived stigma. In addition, age was statistically significantly associated with maladaptive coping. The results suggested an inverse relationship between age and maladaptive coping mechanisms, except for the youngest (and smallest in terms of sample size) age category, 20–29.

**Table 4:** Results of significant variables in the regression models predicting coping

	**Adaptive Coping**		**Maladaptive Coping**	
	Coefficient	95% CI	Coefficient	95% CI
**Age group**				
20–29	-	-	-	-
30–39	0.01	(-2.43, 2.46)	1.41	(0.20, 2.63)
40–49	-0.92	(-3.11, 1.27)	0.11	(-0.93, 1.15)
50–59	0.41	(-1.96, 2.79)	-0.77	(-1.73, 0.20)
60 +	1.33	(-1.58, 4.24)	-0.94	(-1.75, -0.13)
**Work length in years**	-0.01	(-0.09, 0.07)	-0.05	(-0.09, -0.02)
**Shortage of PPE makes me anxious**^**a**^	0.66	(0.06, 1.27)	0.24	(-0.03, 0.52)
**Able to protect family from COVID-19**^**a**^	0.06	(-0.66, 0.79)	-0.49	(-0.91, -0.07)
**Impact of COVID-19 on life**				
Not at all/Somewhat (reference)	-	-	-	-
Very much	2.67	(0.89, 4.46)	1.46	(0.59, 2.33)
**Stigma score**	0.96	(-0.41, 2.34)	1.90	(0.95, 2.84)
**Functional Social Support**	1.38	(0.38, 2.38)	-0.36	(-1.01, 0.28)

^a^Variable measured on a Likert scale with 1 = Strongly Disagree and 5 = Strongly Agree

## Discussion

This study assessed COVID-19 related stress, coping and wellbeing among last responders in the US. We found high levels of stress and anxiety among our respondents. Similarly, approximately 20% of first responders experience clinical levels of anxiety during COVID-19 [18] as compared to 11% of our sample that experienced severe anxiety, and 19% moderate to severe anxiety. A study of the mental health status of Canadian last responders surveyed during the early months of the pandemic also found moderate to severe symptoms of anxiety and depression were common [8]. Additionally, our results highlight high levels of social support, and both adaptive and maladaptive coping mechanisms in the face of the COVID-19 pandemic. Regrettably, our data suggest that last responders also experience significant stigma as a result of their profession, a finding which has also been noted previously [19, 20]. Similarly, first responders experienced stigma during COVID-19 [21].

Several covariates were consistently and significantly related to the three outcomes of wellbeing, anxiety, and stress. Being male, more work length in years, confidence in one’s ability to protect family members from COVID-19, experience of less stigma, and stronger perceptions of social support were associated with less stress, less anxiety, and greater wellbeing. These outcomes align with results from other studies with last responders. A study produced in Belgium seeking to understand compassion fatigue among funeral directors during and following the first wave of COVID-19, found a significant association between secondary trauma and being female [22]. Similarly, pre-COVID-19 studies suggested physical work environment and perceived stress are associated with higher levels of anxiety in female funeral service practitioners [3], while a study of South African mortuary workers found perceived stress to be related to elevated symptoms of depression [23]. In another pre-COVID-19 study, death anxiety was found to decrease with age among U.S. funeral home personnel [4], pairing with our data that longer time working in the field showed a decrease in overall anxiety.

Research among medical staff and first responders during the COVID-19 pandemic also generally confirms our results. Medical staff who were concerned about their own or their family’s safety related to COVID-19 had greater stress [24]. Also, a study of mental health among first responders during COVID-19 found that COVID-19-related worry was associated with higher anxiety [18]. This study found no difference in mental health outcomes by gender, although others have found similarly to ours: female and younger health care workers having more severe psychological symptoms than their counterparts [8, 24, 25]. A systematic review of stigma among first responders found stigma to be associated with depression [21].

Coping was captured by two subscales—adaptive or maladaptive—for each of the variables. Maladaptive coping mechanisms were associated with higher reports of stigma and adaptive coping mechanisms were associated with higher social support. A previous study found social support to be a significant predictor of positive psychological changes in funeral directors [26]. Social support provided to healthcare workers during COVID-19 was associated with less stress and anxiety [24], whereas social support was found to be associated with more distress among healthcare workers in Italy [27]. In addition, although not explicitly assessed in the context of coping, a pre-COVID-19 study [28] also found elevated levels of stress, depression, and anxiety symptoms, as well as high rates of cigarette smoking (35%) and consumption of 4 + alcoholic drinks per occasion when consuming alcohol (17%) among funeral directors, which could represent maladaptive coping strategies. Notably, participants also reported that they often do not get the social support that they need [28].

Although we included a variable measuring excess mortality during the pandemic in the State in which the respondent worked, as a covariate in all the models, it was significantly associated only with wellbeing. This result was unexpected and needs further exploration.

Our study has several limitations. The design is cross-sectional, and therefore any associations are correlational and not causation. In addition, our sample includes last responders from only 43 States, and our sample of respondent were majority white and non-Hispanic, both these factors limit generalizability of findings. This latter limitation was found in a study of mental health of first responders during the COVID-19 pandemic [18]. We also only asked about State where last responders worked, but not whether the location they worked was rural or urban, limiting our ability to conduct more granular place analyses. All the measures used in the survey are self-reported, and therefore could be misreported or biased, limiting validity of our results. Finally, due to our recruitment strategy, we were not able to calculate response rates or determine the representativeness of our sample, overall or by State. Our study also has several strengths. To our knowledge, this is the first national survey of last responders in the US. We also used validated scales to measure wellbeing, anxiety, stress and coping.

Results of this study have implications for future research as well as policy and practice. The study team has recently completed interviews with over 25 last responders. Thematic analysis is ongoing and has provided deep insight into the lived experience of last responders in relation to the COVID-19 pandemic. Interventions to support last responders cope with the stress of the work, to decrease anxiety and enhance wellbeing are critically needed, particularly for younger, and female last responders. These might include low intensity peer-to-peer interventions such as Problem Management Plus [29], tailored to the needs of last responders; or evidence-based interventions for front liners [30] or interventions that have been pilot tested with first responders during the COVID-19 pandemic [31, 32]. In addition, there is a critical need to challenge the community stigma towards last responders by developing a mass media communication campaign directed at the general public [2]. Results could also inform the development of workplace policies to enhance wellbeing among last responders. The National Institute of Occupational Safety and Health (NIOSH) Total Worker Health approach provides such guidance [33]. Finally, our results also suggest the importance of policy responses that value the work of last responders, and acknowledge the risks of their jobs, by prioritizing last responders for vaccination and distribution of PPE in future pandemics, and ensuring provision of mental health support resources.

## Conclusions

Last responders are a critical part of the health care system. They are vital to the comfort and dignity of deceased individuals, and to their families, as they provide compassion, care, and respect at the end of life. Throughout this pandemic, last responders have been frequently ignored and not prioritized for protection and support. Our findings indicate the dangerous effects of this disregard. We urge a greater understanding of, attention to, and deep appreciation of the essential role of last responders in our lives and communities.

### Supplementary Information

**Additional file 1. **Fatalities Management Workers and COVID-19 Stress, Coping and Wellbeing Survey.

## Data Availability

The datasets generated and/or analysed during the current study are not publicly available due to ongoing primary research question analyses but are available from the corresponding author on reasonable request.
