# Supplementary material for: Ignored and distressed: a cross-sectional study of the impact of COVID-19 on last responders
Source: BMC Public Health. 2023 Aug 26;23:1637. doi: 10.1186/s12889-023-16565-z (PMC10463888; doi:10.1186/s12889-023-16565-z)
Supplement: Supplementary file 1 — Additional file 1. Fatalities Management Workers and COVID-19 Stress, Coping and Wellbeing Survey. [file 12889_2023_16565_MOESM1_ESM.docx]

# Fatalities Management Workers and COVID-19 Stress, Coping and Wellbeing Survey

The Coronavirus 19 (COVID-19) pandemic has resulted in a high volume of demand on fatalities management workers across the US. Furthermore, the highly infectious nature of COVID-19 and the social distancing requirements have created conditions that may increase stress for fatalities management workers. This survey aims to assess COVID-19-related stress, coping, and well-being among fatalities management workers.

Please read the instruction to each part of this survey carefully prior to responding. Some of the questions ask specifically about your feelings or behaviors in relation to COVID-19. Others ask about how you respond to situations generally (with or without COVID-19). There are no right or wrong answers to any of the questions in this survey. Respond to each question in terms of what most represents your feelings or behaviors. Every response is the right one for you. The results are invaluable to our understanding of the experiences faced by fatalities management workers.

We understand that this survey may feel somewhat redundant or long. This is part of the research rigor. We appreciate you completing the survey and thank you for your willingness to provide information, as this will allow us to develop interventions, programs, and policies which can support fatalities management workers in situations such as COVID-19 in the future.

Before we start, please create a personal ID using the following guidelines: ·

- First three letters of your mother’s maiden name (for example: BROwn) ·
- First three letters of your city of birth (for example: ATLanta) ·
- Your 2 digit day of birth (for example: 05/04/1990)

In the above example, the personal ID would be broatl04 Please create your Personal ID using the guidelines above:

**Anticipatory Reaction:** The following questions ask about the extent to which the COVID-19 pandemic is or is not leading to specific **concerns about the future:** For the below statement, please select the extent to which you agree or disagree with the statement.

| **Statement** | **Strongly disagree** | **Somewhat disagree** | **Neither disagree nor agree** | **Somewhat agree** | **Strongly agree** |
| --- | --- | --- | --- | --- | --- |
| 1. I feel anxious that I might be exposed to COVID-19. |  |  |  |  |  |
| 2. I find myself thinking that the world is becoming increasingly  dangerous as a result of COVID-19. |  |  |  |  |  |
| 3. I do not feel concerned that COVID-19 will affect my family. |  |  |  |  |  |

| 4. I discuss the possibility of COVID-19 infecting my family and  friends in the future. |  |  |  |  |  |
| --- | --- | --- | --- | --- | --- |
| 5. I have trouble sleeping because of COVID-19. |  |  |  |  |  |
| 6. Thinking about the COVID-19 makes me feel generally  pessimistic about the future. |  |  |  |  |  |
| 7. I feel distressed at the possibility that COVID-19 might infect  either myself or my family and friends in the future. |  |  |  |  |  |
| 8. I search for further information about COVID-19 (e.g., news  stories or information on the Internet). |  |  |  |  |  |
| 9. The possibility of COVID-19 infecting my family or me makes  me angry. |  |  |  |  |  |
| 10. I plan or carry out actions to protect myself or others from COVID-19 (e.g., stocking emergency supplies), |  |  |  |  |  |
| 11. Thinking about COVID-19 makes me feel like my family and I may not be safe. |  |  |  |  |  |
| 12. I feel frightened that COVID-19 may infect me or people I care  about. |  |  |  |  |  |
| 13. I discuss COVID-19 with others (e.g., in person or by social media) to make them aware of possible future risks. |  |  |  |  |  |
| 14. I find myself imagining COVID-19 infecting my family and friends or me. |  |  |  |  |  |
| 15. The possibility of COVID-19 infecting me or people I care about makes me feel tense. |  |  |  |  |  |
| 16. I think of plans to help keep my family and myself safe if COVID-19 happens to us. |  |  |  |  |  |
| 17. Thinking about the possibility of COVID-19 occurring in my life makes it hard to concentrate on daily tasks. |  |  |  |  |  |
| 18. I warn family and friends to avoid places and things that might  put them at risk from COVID-19. |  |  |  |  |  |
| 19. I feel horrified that COVID-19 could infect me or those I care  about in the future. |  |  |  |  |  |
| 20. I find it hard to get on with things I should be doing because I  can’t help thinking about what would happen if COVID-19 infected me or my family. |  |  |  |  |  |

**Difficult moral decisions:** In this section, we are interested in learning about some of the **moral issues** involved in fatalities management work during the COVID-19 pandemic. By “moral,” we mean your own standards of behavior or beliefs about right and wrong. Please respond by selecting the extent to which you agree or disagree with the following statements.

| **Statement**  **During the COVID-19 pandemic …** | **Strongly disagree** | **Somewhat disagree** | **Neither disagree nor agree** | **Somewhat agree** | **Strongly agree** | **Not applicable** |
| --- | --- | --- | --- | --- | --- | --- |
| 1) I saw things that were morally wrong. |  |  |  |  |  |  |
| 2) I am troubled by having witnessed others’ immoral acts. |  |  |  |  |  |  |
| 3) I acted in ways that violated my own moral code or values. |  |  |  |  |  |  |
| 4) I am troubled by having acted in ways that violated my own morals or values. |  |  |  |  |  |  |
| 5) I violated my own morals by failing to do something that I felt I  should have done. |  |  |  |  |  |  |
| 6) I am troubled because I violated my morals by failing to do something that I felt I should have done. |  |  |  |  |  |  |
| 7) I feel betrayed by systems I once trusted. |  |  |  |  |  |  |
| 8) I feel betrayed by supervisors whom I once trusted. |  |  |  |  |  |  |
| 9) I feel betrayed by fellow fatalities management workers whom I once trusted. |  |  |  |  |  |  |

**Sense of control:** These questions ask about the extent to which **you generally** may or may not **feel in control**. For the below statement, please select the extent to which you agree or disagree with the statement.

| **Statement** | **Strongly disagree** | **Somewhat disagree** | **Neither disagree nor agree** | **Somewhat agree** | **Strongly agree** |
| --- | --- | --- | --- | --- | --- |
| 1. I can do just about anything I really set my mind to. |  |  |  |  |  |
| 2. When I really want to do something, I usually find a way to  succeed at it. |  |  |  |  |  |
| 3. Whether or not I am able to get what I want is in my own hands. |  |  |  |  |  |
| 4. What happens to me in the future mostly depends on me. |  |  |  |  |  |
| 5. There is little I can do to change many of the important things in  my life. |  |  |  |  |  |
| 6. I often feel helpless in dealing with the problems of life. |  |  |  |  |  |
| 7. Other people determine most of what I can and cannot do. |  |  |  |  |  |
| 8. What happens in my life is often beyond my control. |  |  |  |  |  |
| 9. There are many things that interfere with what I want to do. |  |  |  |  |  |

| 10. I have little control over the things that happen to me. |  |  |  |  |  |
| --- | --- | --- | --- | --- | --- |
| 11. There is really no way I can solve the problems I have. |  |  |  |  |  |
| 12. I sometimes feel I am being pushed around in my life. |  |  |  |  |  |

**Response to stress:** The questions that follow ask about **the way you generally respond to stress**. For the below statement, please select the extent to which you agree or disagree with the statement.

| **Statement** | **Strongly disagree** | **Somewhat disagree** | **Neither disagree nor agree** | **Somewhat agree** | **Strongly agree** |
| --- | --- | --- | --- | --- | --- |
| 1. I tend to bounce back quickly after hard times. |  |  |  |  |  |
| 2. I have a hard time making it through stressful events. |  |  |  |  |  |
| 3. It does not take me long to recover from a stressful event. |  |  |  |  |  |
| 4. It is hard for me to snap back when something bad happens. |  |  |  |  |  |
| 5. I usually come through difficult times with little trouble. |  |  |  |  |  |
| 6. I tend to take a long time to get over set-backs in my life. |  |  |  |  |  |

**Social Support:** The following questions ask about **your current relationship with friends, family members, coworkers, community members, and others that are close to you**. We know COVID-19 may have affected your answer to some of these questions. Please select the extent to which you agree or disagree that each statement describes your current relationships with other people since the COVID-19 pandemic began.

| **Statement**  **Since the COVID-19 pandemic began …** | **Strongly disagree** | **Somewhat disagree** | **Neither disagree nor agree** | **Somewhat agree** | **Strongly agree** |
| --- | --- | --- | --- | --- | --- |
| 1. I have people who care what happens to me. |  |  |  |  |  |
| 2. I get love and affection. |  |  |  |  |  |
| 3. I get chances to talk to someone about problems at work or with  my home life. |  |  |  |  |  |
| 4. I get chances to talk to someone I trust about my personal or  family problems. |  |  |  |  |  |
| 5. I get chances to talk about money matters. |  |  |  |  |  |
| 6. I get useful advice about important things in life. |  |  |  |  |  |
| 7. When I am sick, there is someone to help me. |  |  |  |  |  |
| 8. I have close relationships that provide me with a sense of  emotional security and well-being. |  |  |  |  |  |
| 9. There is someone I could talk to about important decisions in my  life. |  |  |  |  |  |
| 10. I have relationships where my competence and skills are  recognized. |  |  |  |  |  |

| 11. I feel part of a group of people who share my attitudes and  beliefs. |  |  |  |  |  |
| --- | --- | --- | --- | --- | --- |
| 12. There are people I can count on in an emergency. |  |  |  |  |  |
| 13. I have been satisfied with the social support that I received from  family members. |  |  |  |  |  |
| 14. I have been satisfied with the social support that I received at  work. |  |  |  |  |  |
| 15. I have been satisfied with the social support that I received from  others. |  |  |  |  |  |
| 16. Sometimes I feel socially isolated outside work because of my  occupation. |  |  |  |  |  |
| 17. I am reluctant to tell people what kind of work I do. |  |  |  |  |  |
| 18. People understand the importance of the work I do as a fatalities  management worker. |  |  |  |  |  |
| 19. People value the work I do as a fatalities management worker. |  |  |  |  |  |
| 20. People understand that I also grieve for the cases I work as a  fatalities management worker. |  |  |  |  |  |

**Perceived Stress:** These questions ask about **how you have generally felt** in the past month, as a result of COVID-19 or any other things happening in your life. For the below statements, please select how often you have felt that way in the last month. Options are never, almost never, sometimes, fairly often, very often.

| **Statement** | **Never** | **Almost never** | **Sometimes** | **Fairly often** | **Very Often** |
| --- | --- | --- | --- | --- | --- |
| 1. In the last month, how often have you been upset because of  something that happened unexpectedly? |  |  |  |  |  |
| 2. In the last month, how often have you felt that you were unable to  control the important things in your life? |  |  |  |  |  |
| 3. In the last month, how often have you felt nervous and  “stressed”? |  |  |  |  |  |
| 4. In the last month, how often have you felt confident about your  ability to handle your personal problems? |  |  |  |  |  |
| 5. In the last month, how often have you felt that things were going  your way? |  |  |  |  |  |
| 6. In the last month, how often have you found that you could not  cope with all the things that you had to do? |  |  |  |  |  |

| 7. In the last month, how often have you been able to control  irritations in your life? |  |  |  |  |  |
| --- | --- | --- | --- | --- | --- |
| 8. In the last month, how often have you felt that you were on top of  things? |  |  |  |  |  |
| 9. In the last month, how often have you been angered because of  things that were outside of your control? |  |  |  |  |  |
| 10. In the last month, how often have you felt difficulties were piling  up so high that you could not overcome them? |  |  |  |  |  |

**Feelings since COVID-19 began:** The questions in this part ask **about how you have felt** since the COVID-19 pandemic started. For the below statements, please select how often you have felt that way. Options are never, almost never, sometimes, fairly often, very often.

| **Statement**  **Since the COVID-19 pandemic started…** | **Never** | **Almost never** | **Sometimes** | **Fairly often** | **Very Often** |
| --- | --- | --- | --- | --- | --- |
| 1. I am happy. |  |  |  |  |  |
| 2. I am preoccupied with more than one case or person I  worked/helped. |  |  |  |  |  |
| 3. I get satisfaction from being a fatalities management worker. |  |  |  |  |  |
| 4. I feel connected to others. |  |  |  |  |  |
| 5. I jump or am startled by unexpected sounds. |  |  |  |  |  |
| 6. I feel invigorated after working a case or with those I help. |  |  |  |  |  |
| 7. I find it difficult to separate my personal life from my role as a  fatalities management worker. |  |  |  |  |  |
| 8. I am not as productive at work because I am losing sleep over  traumatic experiences of a case I worked or a person I helped. |  |  |  |  |  |
| 9. I think that I might have been affected by the traumatic stress of  working a case or of those I helped. |  |  |  |  |  |
| 10. I feel trapped by my professional duties as a fatalities  management worker. |  |  |  |  |  |
| 11. Because of my fatalities management work, I have felt "on  edge" about various things. |  |  |  |  |  |
| 12. I like my work as a fatalities management worker. |  |  |  |  |  |
| 13. I feel depressed because of the traumatic experiences of my work as a fatalities management worker. |  |  |  |  |  |

| 14. I feel as though I am experiencing the trauma of someone I  have helped. |  |  |  |  |  |
| --- | --- | --- | --- | --- | --- |
| 15. I have beliefs that sustain me. |  |  |  |  |  |
| 16. I am pleased with how I am able to keep up with the increased  responsibilities and protocols of handling Covid-19 cases. |  |  |  |  |  |
| 17. I am the person I always wanted to be. |  |  |  |  |  |
| 18. My work makes me feel satisfied. |  |  |  |  |  |
| 19. I feel worn out because of my work as a fatalities management  worker. |  |  |  |  |  |
| 20. I have happy thoughts and feelings about those I helped and  how I could help them. |  |  |  |  |  |
| 21. I feel overwhelmed because my case work load seems endless. |  |  |  |  |  |
| 22. I believe I can make a difference through my work. |  |  |  |  |  |
| 23. I avoid certain activities or situations because they remind me  of frightening experiences of the cases I worked or the people I helped. |  |  |  |  |  |
| 24. I am proud of what I can do as a fatalities management worker. |  |  |  |  |  |
| 25. As a result of my work, I have intrusive, frightening thoughts. |  |  |  |  |  |
| 26. I feel "bogged down" by the system. |  |  |  |  |  |
| 27. I have thoughts that I am a "success" as a fatalities management workers. |  |  |  |  |  |
| 28. I can't recall important parts of my work with Covid-19 victims. |  |  |  |  |  |
| 29. I grieve for the cases I worked. |  |  |  |  |  |
| 30. I am a very caring person. |  |  |  |  |  |
| 31. I am happy that I chose to be a fatalities management worker. |  |  |  |  |  |

# Impact of COVID-19 / part I

Has the COVID-19 pandemic led to **any of the following positive changes in your life**? Options are not at all, slightly, moderately, very, extremely.

| **Statement**  **Has the COVID-19 pandemic led to…** | **Not at all** | **Slightly** | **Moderately** | **Very** | **Extremely** |
| --- | --- | --- | --- | --- | --- |
| **…**a strengthening of your relationships with others or your  community. |  |  |  |  |  |
| …thoughts of new possibilities in your life. |  |  |  |  |  |
| …helping you identify personal strengths. |  |  |  |  |  |

| …a personal spiritual change. |  |  |  |  |  |
| --- | --- | --- | --- | --- | --- |
| …an increase in your appreciation of life. |  |  |  |  |  |

# Impact of COVID 19 / Part II

Since the COVID-19 pandemic began, **which of the following work and employment related impacts happened to you**? (check yes or no to all that apply. If the situation does not apply, for example of – for the third statement, you do not have people you supervise, choose ‘not applicable’)

| **Statement** | **Yes** | **No** |
| --- | --- | --- |
| 1. Laid off from job or had to close own practice. |  |  |
| 2. Reduced work hours or furloughed. |  |  |
| 3. Had to lay-off or furlough employees or people I supervised. |  |  |
| 4. Had to continue to work even though in close contact with people who might be infected (e.g., clients,  patients, co-workers). |  |  |
| 5. Spend a lot of time disinfecting at home due to close contact with people at work who might be infected. |  |  |
| 6. Increase in workload or work responsibilities. |  |  |
| 7. Hard time doing job well because of needing to take care of my family members. |  |  |
| 8. Hard time making the transition to home from work. |  |  |
| 9. Provided direct care to people with the disease. |  |  |
| 10. Provided supportive care to people with the disease. |  |  |
| 11. Provided care to people who died as a result of the disease. |  |  |
| 12. Provided supportive care to family members of people who died of the disease. |  |  |
| 13. Had to work long hours with very little rest. |  |  |
| 14. Worked many days with no days off. |  |  |
| 15. Had to work even when I felt exhausted. |  |  |
| 16. Continued to push myself to work even when I felt I was making unsafe decisions. |  |  |
| 17. Hard time making the transition to working virtually. |  |  |
| 18. I have been less careful at work. |  |  |

**Coping strategies**: These items deal with ways you've been coping with the stress in your life since the COVID-19 pandemic began. There are many ways to try to deal with problems. These items ask what you've been doing to cope with this one. For the below statements, please select **how often you have engaged in that coping strategy** since the COVID-19 pandemic started. Don't answer on the basis of whether it seems to be working or not—just whether or not you're doing it. If any of the statements do not apply to you, for example for the last three statements, if you are not currently in an intimate relationship, do not have a partner at home, or do not have children, please select 'not applicable.'

| **Statement**  **Since the COVID-19 pandemic started …** | **I haven’t been doing this at all** | **I’ve been doing this a little bit** | **I’ve been doing this a medium**  **amount** | **I’ve been doing this a**  **lot** | **Not applicable**  **(N/A)** |
| --- | --- | --- | --- | --- | --- |

| 1. I’ve been turning to work or other activities  to take my mind off things |  |  |  |  |  |
| --- | --- | --- | --- | --- | --- |
| 2. I’ve been concentrating my efforts on doing  something about the situation I’m in. |  |  |  |  |  |
| 3. I’ve been saying to myself “this isn’t real”. |  |  |  |  |  |
| 4. I’ve been using alcohol or other drugs to  make myself feel better. |  |  |  |  |  |
| 5. I've been getting emotional support from  others. |  |  |  |  |  |
| 6. I’ve been giving up trying to deal with it. |  |  |  |  |  |
| 7. I’ve been taking action to try to make the  situation better. |  |  |  |  |  |
| 8. I’ve been refusing to believe that it has  happened. |  |  |  |  |  |
| 9. I’ve been saying things to let my unpleasant  feeling escape. |  |  |  |  |  |
| 10. I’ve been getting help and advice from other  people. |  |  |  |  |  |
| 11. I’ve been using alcohol or other drugs to  help me get through it. |  |  |  |  |  |
| 12. I’ve been trying to see it in a different light,  to make it seem more positive. |  |  |  |  |  |
| 13. I’ve been criticizing myself. |  |  |  |  |  |
| 14. I’ve been trying to come up with a strategy  about what to do. |  |  |  |  |  |
| 15. I’ve been getting comfort and understanding from someone outside of my normal  relationship. |  |  |  |  |  |
| 16. I’ve been giving up my attempt to cope. |  |  |  |  |  |
| 17. I’ve been looking for something good in what is happening. |  |  |  |  |  |
| 18. I’ve been making jokes about it. |  |  |  |  |  |
| 19. I’ve been doing something to think about it less, such as going to movies, watching TV, reading, daydreaming, sleeping, or  shopping. |  |  |  |  |  |

| 20. I’ve been accepting the reality that COVID-  19 has changed how I feel about my safety and health. |  |  |  |  |  |
| --- | --- | --- | --- | --- | --- |
| 21. I’ve been expressing my negative feelings. |  |  |  |  |  |
| 22. I’ve been trying to find comfort in my  religious or spiritual beliefs. |  |  |  |  |  |
| 23. I’ve been trying to get advice or help from  other people about what to do. |  |  |  |  |  |
| 24. I’ve been learning to live with it. |  |  |  |  |  |
| 25. I’ve been thinking hard about what steps to  take. |  |  |  |  |  |
| 26. I’ve been blaming myself for things that happened. |  |  |  |  |  |
| 27. I’ve been praying or meditating. |  |  |  |  |  |
| 28. I’ve been making fun of the situation. |  |  |  |  |  |
| 29. I’ve been limiting autopsies when possible. |  |  |  |  |  |
| 30. I’ve been limiting exposure to personal  effects when possible. |  |  |  |  |  |
| 31. I’ve been lessening my exposure to the  hands and face of the deceased. |  |  |  |  |  |
| 32. I’ve been reaching out to fellow fatalities management workers who have military, mass casualty, or more experience in general  for guidance and tips. |  |  |  |  |  |
| 33. I’ve been recording emotional impacts and  thoughts (e.g., journaling, voice recording, or self-talk). |  |  |  |  |  |
| 34. I’ve been reflecting on the importance of the role I have taken to help the families of  deceased individuals. |  |  |  |  |  |
| 35. I’ve been remembering that my work is important for the loved ones of the  decedents and for my community. |  |  |  |  |  |
| 36. I have considered leaving my existing  intimate relationship. |  |  |  |  |  |

| 37. I have argued more with my partner at  home. |  |  |  |  |  |
| --- | --- | --- | --- | --- | --- |
| 38. I have had less patience with my children. |  |  |  |  |  |

**General Feelings-1**: These questions ask about **general** feelings you may have been experiencing recently. Over the last 2 weeks, how often have you been bothered by the following problems? Options are not at all, several days, more than half the days, nearly every day.

| **Statement/problem** | **Not at all** | **Several days** | **More than half the days** | **Nearly every day** |
| --- | --- | --- | --- | --- |
| 1. Feeling nervous, anxious or on  edge. |  |  |  |  |
| 2. Not being able to stop or control  worrying. |  |  |  |  |
| 3. Worrying too  much about different things. |  |  |  |  |
| 4. Trouble relaxing. |  |  |  |  |
| 5. Being so restless  that it is hard to sit still. |  |  |  |  |
| 6. Becoming easily annoyed or irritable. |  |  |  |  |
| 7. Feeling afraid as if something awful might  happen. |  |  |  |  |

**General feelings - 2:** Please indicate for each of the five statements which is closest to how you have been feeling over the last two weeks.

| **Statement**  **Over the last two weeks …** | **At no time** | **Some of the time** | **Less than half of the**  **time** | **More than half of the**  **time** | **Most of the**  **time** | **All of the time** |
| --- | --- | --- | --- | --- | --- | --- |
| 1. I have felt cheerful and in good spirits. |  |  |  |  |  |  |
| 2. I have felt calm and relaxed. |  |  |  |  |  |  |

| 3. I have felt active and vigorous. |  |  |  |  |  |  |
| --- | --- | --- | --- | --- | --- | --- |
| 4. I woke up feeling fresh and rested. |  |  |  |  |  |  |
| 5. My daily life has been filled with things that interest  me. |  |  |  |  |  |  |

**Self care:** In the past month, to **cope with the stress of the COVID-19 pandemic and social distancing and isolation**, are you doing any of the following? Please select all that apply.

| **Statement** | **Yes** | **No** |
| --- | --- | --- |
| Intentionally taking breaks from watching, reading, or listening to news stories, including social  media. |  |  |
| Increasing watching, reading, or listening to news stories, including social media. |  |  |
| Taking care of your body, such as taking deep breaths, stretching, or meditating. |  |  |
| Engaging in healthy behaviors like trying to eat healthy, well-balanced meals, exercising regularly,  getting plenty of sleep, or avoiding alcohol and drugs. |  |  |
| Making time to relax. |  |  |
| Connecting with others, including talking with people you trust about your concerns and how you are  feeling. |  |  |
| Contacting a healthcare provider. |  |  |
| Smoking more cigarettes or vaping more. |  |  |
| Drinking alcohol more than usual. |  |  |
| Using prescription drugs to calm down (like valium, etc.) more than usual. |  |  |
| Using non-prescription drugs more than usual. |  |  |
| Using cannabis or marijuana more than usual. |  |  |
| Eating high fat or sugary foods more than usual. |  |  |
| Cutting or self-injury more than usual. |  |  |
| Over exercise more than usual. |  |  |
| Eating more food than usual. |  |  |
| Eating less food than usual. |  |  |
| Contacted a mental health counselor/provider. |  |  |
| Contacted a clergyman or spiritual care provider. |  |  |

# I believe I can protect myself from COVID-19

- Strongly disagree
- Somewhat disagree
- Neither disagree nor agree
- Somewhat agree
- Strongly agree

# I believe I can protect my family from COVID-19

- Strongly disagree
- Somewhat disagree
- Neither disagree nor agree
- Somewhat agree
- Strongly agree

# How much has the COVID-19 pandemic impacted your day-to-day life?

1. Not at all
2. Somewhat
3. Very Much

**General questions –** In this final section, we are asking some general questions about you.

1. What is your occupation within the field of Fatalities Management? (check all that apply)
   1. Funeral Service Practitioner
   2. Funeral home staff (unlicensed)
   3. Medical Examiner/Coroner
   4. Medical Examiner/Coroner Investigator
   5. Pathologist
   6. Medical Examiner/Coroner Investigator staff (unlicensed)
   7. Crematory Operator
   8. Hospital Decedent Care Specialist/Morgue Attendant/Autopsy Assistant
   9. Funeral Service Intern/Student
   10. Medical Examiner/Coroner Intern/Student
   11. Other (please specify)
2. How long have you worked in fatalities management? Please fill in both years, and months. So if you have worked in fatalities management for only 4 months, please put 0 in the ‘years’ box, and 4 in the ‘months’ box. If you have worked in fatalities management 6 years, and 2 months, please put 6 in the ‘years’ box, and 2 in the ‘months’ box. If you can approximate the years you have been working in fatalities management but not the months, please put the number of years in the 'years' box, and 0 in the 'months' box.
   1. Years
   2. months
3. What state do you currently work in as a fatalities management worker?
   1. (Drop down menu of all the States)
4. Sometimes, people feel as though they are **treated differently** than others because they are fatalities management workers. In your day-to-day life, how often do any of the following things happen to you?

| Statement | Often | Sometimes | Rarely | Never |
| --- | --- | --- | --- | --- |
| You are treated with less courtesy than other people are. |  |  |  |  |
| You are treated with less respect than other people are. |  |  |  |  |
| You receive poorer service than other people at restaurants or stores. |  |  |  |  |
| People act as if they think you are not smart. |  |  |  |  |
| People act as if they are afraid of you. |  |  |  |  |
| People act as if they think you are dishonest. |  |  |  |  |
| People act as if they’re better than you are. |  |  |  |  |
| You are called names or insulted. |  |  |  |  |
| You are threatened or harassed. |  |  |  |  |

1. As part of your work as a fatalities management worker, how much experience have you had with handling persons who died as a result of infectious and/or dangerous pathogens before the COVID-19 pandemic?
   1. A lot
   2. Some
   3. Not at all
2. As part of your work as a fatalities management worker, have you **ever** been involved in caring for the dead in a mass fatalities situation (mass violence, plane crashes, natural disaster, war, etc) before the COVID-19 pandemic?
   1. Yes
   2. No
3. Since the COVID-19 pandemic was first identified in the USA, have you had to care for persons who died of COVID-19, or possible COVID- 19?
   1. Yes, approximately how many deaths have you handles that were confirmed or possible COVID-19 regardless of whether they were later identified as negative?
   2. No
4. If yes to question #7, Where did you pick up the remains(s)? Check all that apply.
   1. I did not have to pick up the remains
   2. At the hospital
   3. At a nursing home
   4. At the person’s home
   5. Other (please specify)
5. If yes to question #7, how often were family members able to be present for the burial/cremation/disposition?
   1. Never
   2. Rarely
   3. Often
   4. Most of the time
6. If rarely or never to question #9, did you feel an added personal responsibility to ensure burial/cremation/disposition was done with dignity, the same amount of burden, or less burden?
   1. More burden
   2. Same amount of burden
   3. Less burden
7. How often did you work with a COVID-19 death where the individual was listed as unknown or unclaimed by anyone?
   1. Never
   2. Rarely
   3. Often
   4. Most of the time
8. If most of the time, often, or rarely to question # 11, did you feel an added personal responsibility to ensure the burial/cremation/disposition was done with dignity, the same amount of burden, or less burden?
   1. More burden
   2. Same amount of burden
   3. Less burden
9. To what extent do you agree or disagree with these statements about personal protective equipment (PPE)

| Statement | Strongly agree | Agree | Neither agree nor disagree | Disagree | Strongly disagree | Not  applicable (N/A) |
| --- | --- | --- | --- | --- | --- | --- |
| I had enough personal protective equipment to safely protect me when I cared for all the  individuals who died from COVID-19 pandemic. |  |  |  |  |  |  |
| I did not use PPE when I felt I should because of  shortages in PPE. |  |  |  |  |  |  |
| I did not use PPE when I felt I should because of  social pressure not to do so. |  |  |  |  |  |  |
| The shortage of PPE makes me anxious. |  |  |  |  |  |  |

1. Has the COVID-19 pandemic made your job in fatalities management harder, the same, or easier?
   1. Harder
   2. The same
   3. Easier
2. To what extent has the COVID-19 pandemic made you think of changing professions (leaving the fatalities management workforce)?
   1. Not at all
   2. Somewhat
   3. A lot
3. Do you have any of the following underlying conditions: (select all that apply)
   1. Asthma
   2. Chronic lung disease
   3. Chronic heart disease
   4. Diabetes
   5. Chronic kidney disease
   6. Cancer in the past year
   7. Immunosuppressive condition
   8. Other, specify
4. Has anyone in your immediate family been infected with COVID-19?
   1. Yes
   2. No
   3. Unsure
   4. Prefer not to answer
   5. Not applicable (N/A)
5. Have you been infected with COVID 19?
   1. Yes, I became infected at my job
   2. Yes, but I did NOT become infected at my job
   3. Yes, but I do not know where I was infected
   4. No
   5. I am unsure if I have been infected
   6. Prefer not to answer
6. Have you tried to get a COVID-19 test and were not able to?
   1. Yes
   2. No
7. What is your sex:
   1. Male
   2. Female
   3. Other
   4. Prefer not to answer
8. What is your age in years:
   1. <20 years
   2. 20-29 years
   3. 30-39 years
   4. 40-49 years
   5. 50-59 year
   6. 60 years or older
   7. Prefer not to answer
9. Are you Hispanic or Latino?
   1. Yes
   2. No
   3. Prefer not to answer
10. Which race(s) are you? Check all that apply.
    1. Asian
    2. Pacific Islander
    3. Native Hawaiian
    4. American Indian/Alaskan Native
    5. White
    6. Black/African American
    7. Other (specify)
    8. Prefer not to answer
11. What language are you most comfortable speaking?
    1. English
    2. Other Please specify
    3. Prefer not to answer
12. How many family members, **including yourself**, do you currently live with? If you live alone, you would answer 1. If you live with others, count all others and yourself.
    1. Number:
    2. Prefer not to answer
13. What is the highest level of school that you have completed?
    1. Less than high school degree
    2. High school diploma of GED
    3. Technical degree (2 years)
    4. Undergraduate degree (4 year)
    5. Graduate degree
    6. Prefer not to answer
14. What is your current work situation? (Pick one answer that best describes your situation)
    1. Unemployed and seeking work
    2. Unemployed but not seeking work (student, retired, primary care giver, other)
    3. Part time or temporary work
    4. Full time work
    5. Full time self employed
    6. Prefer not to answer
15. What is your main insurance?
    1. None/uninsured
    2. Medicaid/Medicare
    3. Other public insurance
    4. Private insurance
    5. Prefer not to answer
16. From which organization did you receive the request to complete this survey? (Check all that apply)
    1. National Association of Medical Examiners (NAME) (1)
    2. International Association of Coroners and Medical Examiners (IACME) (2)
    3. American Board of Medicolegal Death Investigators (ABMDI) (3)
    4. Council of Funeral Association Executives (CFAE) (4)
    5. National Funeral Directors Association (NFDA) (5)
    6. Cremation Association of North America (CANA) (6)
    7. International Cemetery, Cremation and Funeral Association (ICCFA) (7)
    8. American Board of Funeral Service Education (ABFSE) (8)
    9. Other organization (9)
    10. I am not a member of any of the above associations. I received the request to complete the survey from a colleague. (10)

The work you are doing is important and can cause stress and influence your mental health. If you need to talk to someone, please reach out to the US department of Health and Human Services Disaster Distress Helpline that provides 24/7, 365-day-a-year crisis counseling and support to people experiencing emotional distress related to natural or human-caused disasters, including COVID-19**. Call 1-800-985-5990 or text TalkWithUs to 66746.** Please click to the next page to let us know if you would like to participate in a follow-up interview and/or survey..

1. We hope to follow-up this survey in two ways:
   1. We will be following up this survey with interviews with fatalities management workers to understand their experiences in much more detail. Interviews will likely begin sometime in early Fall.
   2. We will be following up in 6 months with this same survey to assess any changes.

If you are willing to participate in either a or b or both, please click here. This will take you to another page to list your name and email. That page if not linked to this one so your answers to this survey remain completely anonymous.

If you are unwilling to participate in either a or b, then click here to end.
